# Comparing calcium influx with high-frequency stimulation and burst stimulation LTP protocols

**DOI:** 10.1186/1471-2202-15-S1-P140

**Published:** 2014-07-21

**Authors:** Ximing Li, William R Holmes

**Affiliations:** 1 Department of Biological Sciences, Neurosciences Program, Ohio University, Athens, OH 45701, USA

## 

Two popular protocols for inducing LTP in CA1 pyramidal cells are high-frequency stimulation (HFS), typically continuous 100 Hz tetanization for 1 second, and burst stimulation with 4 pulses at 100 Hz repeated at 200 ms intervals. NMDA blockers prevent LTP with burst stimulation, but some NMDA-independent LTP remains with HFS. Conversely, the L-channel antagonist nifedipine strongly inhibits LTP following HFS, but has a relatively small effect on LTP induced by burst stimulation [1]. These different effects are likely to be produced by different levels of calcium influx from different sources caused by different voltage responses generated with the two protocols. Here we sought to compare calcium influx at spines and the cell body produced by these two protocols quantitatively by using computational models of a CA1 pyramidal cell with full morphology.

With continuous tetanization it has been observed that EPSPs sum and produce a large depolarization that triggers action potentials early in the train, but later in the train EPSPs are smaller and fail to produce action potentials even though voltage remains elevated [1]. To account for this EPSP depression in the models, we incorporated data for the probability of vesicle release at synapses at each position in a long HFS train [2]. For burst stimulation, it has been observed that each burst reliably produces 1-4 action potentials, but voltage between bursts returns to a lower level than with HFS. For the probability of release at each synapse for each position in the burst, we used the data for probability of release for the first four pulses in the HFS train. No attempt was made to include facilitation sometimes seen during burst stimulation. The total number of stimuli was 80 with each protocol. Calcium influx through NMDA receptor channels was computed at several dendritic spines and calcium influx through L-type calcium channels was computed at the soma.

We found that total calcium influx at the soma was considerably larger with burst stimulation than with a continuous tetanus and was directly related to the number of action potentials produced by each protocol. However calcium influx with continuous tetanization was larger early in the train causing the average soma calcium concentration change for the first few hundred milliseconds to be up to 2-fold larger than with burst stimulation. At spines calcium influx was also larger at early times with continuous tetanization, but again total calcium influx was considerably larger with burst stimulation. Curiously, we occasionally saw spikelets of 10s of mV generated in the dendrites with continuous tetanization, but these did not propagate successfully to affect soma voltage. Initial spikes or spikes generated after a quiet period tended to be generated at the initial segment, but the site of action potential generation of subsequent spikes moved into the dendrites.

The larger total calcium influx seen with burst stimulation is consistent with the observation that this is a more effective protocol than continuous tetanization for inducing LTP. How the differences in calcium signals observed at spines and the soma with these two different protocols affect LTP induction or explain the different actions of L-channel and NMDA blockers on LTP induction with these protocols awaits further study.

## References

[B1] Grover LM Kim E Cooke JD Holmes WR LTP in hippocampal area CA1 is induced by burst stimulation over a broad frequency range centered around delta Learn Mem 2009 15 69 81 10.1101/lm.1179109 19144965 PMC2632851

[B2] Kim E Owen B Holmes WR Grover LM Decreased afferent excitability contributes to synaptic depression during high-frequency stimulation in hippocampal area CA1 J Neurophysiol 2012 15 1965 1976 10.1152/jn.00276.2011 22773781 PMC3544996

